# An Extremely Rare Case of Isolated Aortic Valve Prolapse Causing Aortic Regurgitation in the Absence of Any Other Pre-existing Cardiovascular or Non-cardiovascular Condition

**DOI:** 10.7759/cureus.46144

**Published:** 2023-09-28

**Authors:** Aayushi Singh, Kanishk V Khurana, Jayanth Kumar, Gajendra Agrawal, Akash Lohakare, Rahul Gutte, Anuj Chaturvedi

**Affiliations:** 1 Cardiology, Jawaharlal Nehru Medical College, Datta Meghe Institute of Higher Education and Research, Wardha, IND; 2 Medicine, Jawaharlal Nehru Medical College, Datta Meghe Institute of Higher Education and Research, Wardha, IND

**Keywords:** prolapsing right coronary and non-coronary cusp, trileaflet aortic valve, transthoracic and transesophageal echocardiography, aortic regurgitation, isolated aortic valve prolapse

## Abstract

In this case report, we present an extremely rare case of isolated aortic valve prolapse causing aortic regurgitation having no association with any comorbid conditions that are commonly seen with aortic valve prolapse. A 27-year-old female patient presented with chief complaints of dyspnea on exertion (New York Heart Association grade III) for 20 days, decreased appetite for 15 days, and a history of significant weight loss for one and a half years. Transthoracic and transesophageal echocardiography revealed a trileaflet floppy aortic valve with prolapsing non-coronary and right coronary cusps, associated with moderate aortic regurgitation. The incidence of aortic valve prolapse is roughly around 1%. Exceptionally, very few cases of isolated aortic valve prolapse with moderate-to-severe aortic regurgitation without any associated pathology have been reported to date.

## Introduction

When the aortic valve structures are pulled or pushed down below the imaginary line connecting the sites where the valvular leaflets join to the annulus, i.e., the aortoventricular junction proximally and sinotubular junction distally, showing a backward bowing in the left ventricle (LV), this condition is referred to as aortic valve prolapse. The most often reported consequence of aortic valve prolapse is aortic regurgitation (AR). AR is a condition in which blood streams backward from the lumen of the aorta into the LV during the diastolic phase due to improper closure of the leaflets of the aortic valve. The incidence of aortic valve prolapse is around 1%. Exceptionally, very few cases of isolated aortic valve prolapse with moderate-to-severe AR without any associated pathology have been reported to date. According to one study, 30% of patients with a prolapse of the aortic valve had a bicuspid valve and an aortic valve prolapse in a non-bicuspid valve was mostly co-existent with a mitral valve prolapse. With further studies, more concomitant etiologies have been found to be linked with aortic valve prolapse which include post-inflammatory, degenerative, traumatic, infectious, and idiopathic or unknown causes [1,2]. However, here, we present a very rare case of a 27-year-old female with an isolated aortic valve prolapse causing moderate AR in the absence of any other cardiovascular or non-cardiovascular condition.

## Case presentation

**Figure 1:**
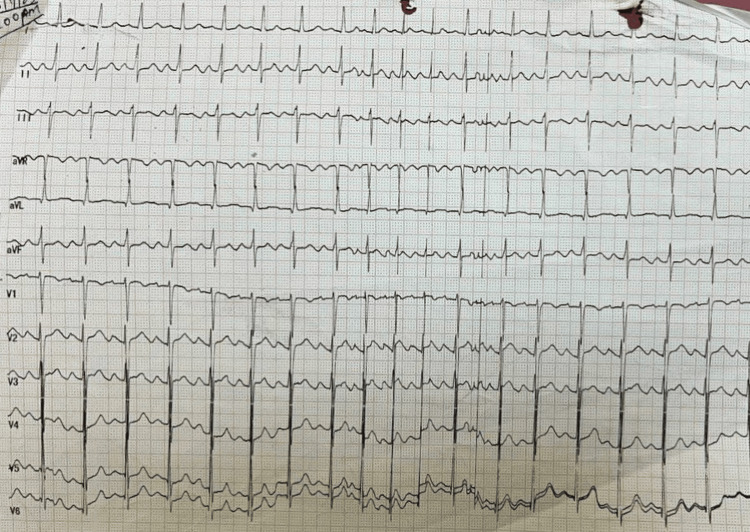
ECG showing sinus tachycardia and left ventricular hypertrophy. The above image has been taken by the authors.

A 27-year-old female patient presented at the cardiology department of a rural tertiary care hospital with chief complaints of dyspnea on exertion (New York Heart Association grade III) for 20 days, decreased appetite for 15 days, and a history of significant weight loss for one and a half years. She had no medically relevant history and had not undergone any surgeries before. Neither had she been on any medications before this visit. There was no history of chest pain, palpitations, syncope, fever, or abdominal pain. She was a non-smoker with no history of cardiovascular diseases in the family. The patient was informed of having been diagnosed with valvular heart disease five years ago but was not on any regular medications. There was no history of rheumatic fever, marfanoid phenotype, or infective endocarditis. Upon general examination, her pulse rate was 128 beats per minute, her blood pressure was 130/90 mmHg, and her respiratory rate was 12 breaths per minute. Cardiac examination showed a tapping apical impulse present in the sixth intercostal space with the presence of parasternal heaving and suprasternal pulsations upon palpation. Upon auscultation, s1 and s2 were heard along with a systolic murmur. An ECG was done which showed sinus tachycardia and left ventricular hypertrophy (Figure 1).

**Figure 2:**
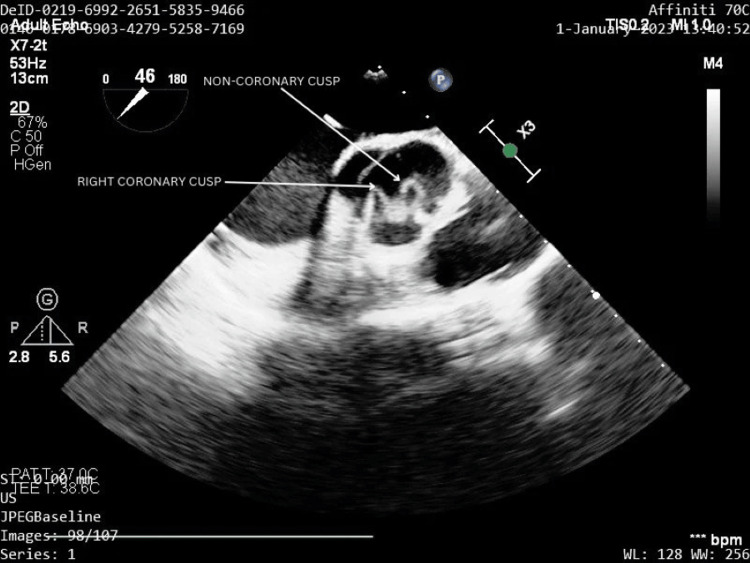
Prolapse of the non-coronary cusp and the right coronary cusp of the aortic valve seen on transesophageal echocardiography (marked with a white arrow). The above image has been taken by the authors.

**Figure 3:**
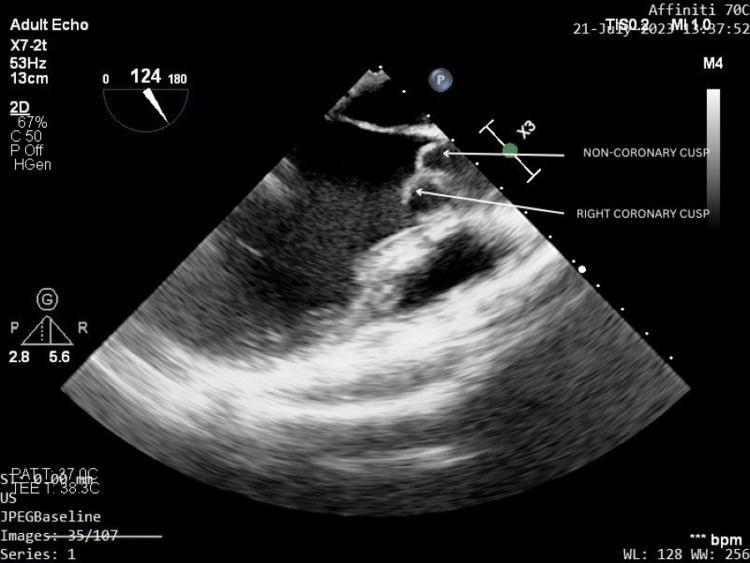
Prolapsed non-coronary and right-coronary cusps of the aortic valve visible on a 120-degree long-axis view of transesophageal echocardiography. The above image has been taken by the authors.

**Figure 4:**
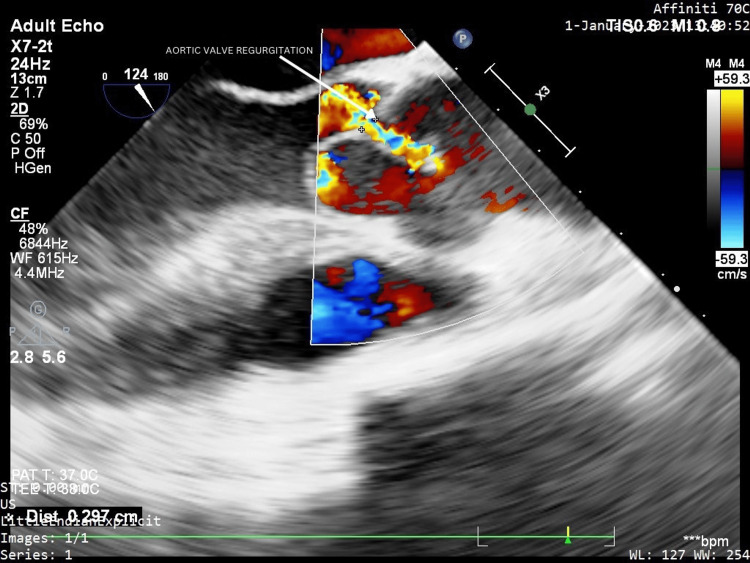
Aortic regurgitation visible on color Doppler transesophageal echocardiography. The above image has been taken by the authors.

A transthoracic echocardiography was done that displayed a trileaflet aortic valve with floppy cusps. Hence, confirmatory transesophageal echocardiography (TEE) was performed for better visualization of the aorta that revealed the aortic valve to have prolapsing non-coronary and right coronary cusps, associated with moderate AR. The aortic root diameter was found to be 20 mm. There was no derangement of other valvular leaflets, i.e., the left coronary cusp of the aortic valve, mitral valve, tricuspid valve, and pulmonary valve appeared to be normal. Her ejection fraction was found to be 55%. There were no visible vegetations of the valve leaflets and no pericardial effusion or clot. The various images of the TEE showing prolapse of the non-coronary cusp and right coronary cusps of the aorta and AR are depicted in Figures 2-4.

The patient was medically managed in the hospital with tablet Met xl 25 mg (metoprolol) and tablet Lasix 20 mg (furosemide) which was indicated due to mild pulmonary congestion as the patient had left heart failure because of moderate AR. The patient was advised further hospital stay for extensive management but her relatives requested discharge due to monetary issues. She was told to revisit for a repeat 2D echocardiography after six months.

## Discussion

The anatomical picture of the aortic valve is trileaflet in an individual with no anomalies, consisting of a right, left, and non-coronary cusp. However, in some cases of congenital malformations, where one or more cusps get fused during embryological development, a unicuspid or bicuspid aortic valve can be present [3]. In very rare scenarios, there can also be a presence of quadricuspid aortic valve congenitally [4]. Of all the types of aortic valves based on the number of leaflets, prolapse is more prevalent in the bicuspid variety, wherein, the anterior leaflet prolapse is notably more common than the posterior leaflet [5,6]. However, in an aortic valve consisting of all three leaflets, the prolapse of the right or non-coronary cusps occurs more often than that of the left coronary cusp, which is also seen in our case report.

Cusp prolapse in the aortic valve can be solitary, with a single or multiple leaflet prolapsing regardless of no alterations in the aortic root, in relation to ascending aortic dilatation, or as the outcome of surgery such as aortic valve-sparing surgeries [7]. There can be various etiologies associated with aortic valve cusp prolapse. These include post-inflammatory, degenerative, traumatic blunt trauma to the chest, infectious, and idiopathic, or unknown causes. Prolapse can be associated with aortic root pathology such as root dilatation, most often seen in Marfan’s syndrome. Due to this, the cusps become overextended and later detach, as in acute dissection [8]. This phenomenon has also been seen in patients with rheumatoid arthritis and congenital bicuspid aortic valve, as mentioned before. A few cases of isolated aortic valve prolapse which is rare have also been reported in patients with syphilitic aortitis [9]. The other cardiac pathologies that pose a risk for developing aortic valve prolapse are infective endocarditis, rheumatic heart disease, hypertrophic cardiomyopathy, severe mitral regurgitation, and mitral valve prolapse, all of which have been well documented by a study published by Shapiro et al. [8]. Aortic valve prolapse has also been widely observed as a late feature in some cases of ventricular septal defect (more frequently with the subpulmonic type) [10]. One can also note that the participation of other valves, i.e., tricuspid (43.3%) and aortic (10%), is a common occurrence in patients suffering from mitral valve prolapse [11]. In a study by Vasan et al., none of the patients without active carditis had aortic valve prolapse [12]. Thus, it is very rare to have a presentation of isolated aortic valve prolapse in the absence of any other associated condition. As our patient had a chronic history of diagnosis of aortic valve prolapse with no other signs and symptoms to support any of the above-mentioned conditions, we come to an understanding that it is an extremely rare case of isolated aortic valve prolapse leading to moderate AR and is very likely congenital or idiopathic due to absence of any other cardiac or extracardiac findings.

The prolapse of the aortic valves can be best diagnosed on a TEE on a mid-esophageal long-axis view of the aortic valve but also requires analysis by surgical inspection. According to Boodhwani et al., the TEE appearance of cusp prolapse shows a reverse or eccentric aortic regurgitant jet opposite to the direction of prolapsing cusp seen in Doppler echocardiography (normally the jet is central), valve leaflets beneath the level of the annulus of the aorta at the time of diastole, reduced length of coaptation (a measure of overlap between two leaflets) of the aortic leaflet, and the emergence of a fibrous band transversely on the cusp which is prolapsing, both in short and long-axis views, aids in confirming the diagnosis of aortic valve prolapse and localizing its site. This is particularly helpful in cases when the left coronary or non-coronary posterior cusp is prolapsed. The short-axis view also shows an asymmetrical root of the aorta along with redundant tissues. Prolapse of more than one cusp is identified by meticulous echocardiographic evaluation of the movements of every cusp at the time of diastole and is usually linked with a decreased level of cusp coaptation [13,14].

The criteria for diagnosing aortic valve prolapse involves looking for the retardation of the aortic valve cusps or their point of coaptation toward the ventricle on the left side in the parasternal long-axis view during diastole. Clear visualization of the aortic valve as well as the structures around it, such as the interventricular septum and anterior cusp of the mitral valve, all positioned along the entire length of the root of the aorta and left ventricular outflow tract, are necessary for adequate aortic valve assessment in TEE. The Woldow et al. approach was used to grade aortic valve prolapse. This is how the severity was rated: 1+ for a minor descending displacement or dipping of the coaptation point causing the cusps to straighten during diastole, 2+ for significant bowing below the level of aortic annulus during diastole, and 3+ for the complete protuberance of one or multiple cusps into the left ventricular area during the diastolic phase [15].

As the aortic valve cusp prolapse as a solitary defect is not that prevalent globally, there is insufficient data results of valve repair over the course of time as well as a lack of understanding of preoperative and intraoperative identification of the prolapsing cusps. Thus, despite the availability of repair techniques such as free margin plication, free margin resuspension using 7-0 polytetrafluoroethylene suture, and triangular resection technique to get rid of broadly solidified and fibrotic tissues of cusp from a central portion, total replacement of the aortic valve remains the preferred choice of treatment for almost all patients suffering from severe AR due to cusp prolapse [13].

## Conclusions

Despite its extreme rarity, aortic valve prolapse with an absence of any other pre-existing cardiovascular or non-cardiovascular condition can cause AR in a patient. It is best early diagnosed with the help of a TEE. Recognizing aortic valve prolapse holds significance as it opens the door to potential valve repair and replacement.
